# Supplementary material for: Leading from the bottom: The clinical leaders roles in an HIV primary care facility in Eldoret, Kenya
Source: PLoS One. 2024 May 31;19(5):e0302066. doi: 10.1371/journal.pone.0302066 (PMC11142606; doi:10.1371/journal.pone.0302066)
Supplement: S1 Fig — (DOCX) [file pone.0302066.s001.docx]

**S1_Fig. 1 AMPATH-MTRH clinical management flow chart**

**WAITING AREA**

- Patients wait to be attended
- Psychosocial talks given by psychosocial team

**RECORDS DESK**

- New patients are registered and assigned clinic ID

**OUTREACH DESK**

- Retrieval of patients’ medical file
- Patient locator information obtained
- Patient appointment data confirmed

**NURSE STATION**

- Patients triaged & vital signs taken by a nurse

**PSYCHOSOCIAL SUPPORT**

- Patients counselled on HIV care by psychological team

**CLINICAL ROOM**

- Patient health assessed and treatment plan created by clinical officer

**PSYCHOSOCIAL SUPPORT**

- Patients’ nutrition assessed by a nutritionist

**PSYCHOSOCIAL SUPPORT**

- Patients’ socio-economic needs assessed by a social worker

**PHARMACY**

- Patient drugs dispensed

**EXIT**

- Patient leaves the clinic
