# Supplementary material for: Leading from the bottom: The clinical leaders roles in an HIV primary care facility in Eldoret, Kenya
Source: PLoS One. 2024 May 31;19(5):e0302066. doi: 10.1371/journal.pone.0302066 (PMC11142606; doi:10.1371/journal.pone.0302066)
Supplement: S1 Table — (DOCX) [file pone.0302066.s002.docx]

**S2_Table. 1 Codebook with minimal data**

| Name | Description | Files | References |
| --- | --- | --- | --- |
| Importance | Importance of clinical leadership in a healthcare system | 22 | 188 |
| Role of a clinical leader | Description of the role of a clinical leader in a healthcare system quote(s) | 22 | 165 |
| Appraise staff and accountable | “Giving the technical know-how and also doing regular assessment of the needs of the service provider and the patients and prioritizing the needs and intervening in accordance to availability of the resources to meet the required needs. And above all, to meet the program objectives.  So he is basically the driver of the objectives of the project” (Nurse)  “Another thing is representation of that unit because in as much as you are working as a team at that place, this leader at some point will have to be accountable for the area in which they are working on so that in case of any issues, you don't always have to fault others. So this particular leader is the one who will carry the burden of being accountable and being responsible for that particular area” (Clinical Officer) | 3 | 3 |
| Assign roles and duties to other staff | “Definitely there will be roles and assigning of roles so that each you meet your objectives of your day which will at the end of the day provide quality health care to our clients and restore their confidence in our clinic” (Clinical Officer) | 7 | 9 |
| Organize and attend meetings | “He also organizes routine meetings; routine meetings at the clinic level with the staff to evaluate the data, to also check for matters arising, any issues” (Clinical Officer) | 5 | 6 |
| Coordinate the day to day activities of the department | “I would say majorly it is coordination; he coordinates the activities and he is the link between the higher management and the client. He coordinates all the activities and the services within the department” (Nurse) | 9 | 13 |
| Ensure availability of resources | “Each and everyday supplies are there… one, to ensure adequate staffing; that is that the staff are enough and the supplies” (Nurse) | 9 | 11 |
| Ensure enough staffing | “Each and everyday supplies are there… one, to ensure adequate staffing; that is that the staff are enough and the supplies” (Nurse) | 2 | 2 |
| Ensure quality services to the patient | “A leader in a healthcare system ensures that our clients are given quality service. For example, a leader ensures that his team or her team are on duty, and are timely with teamwork, so in the long run we give quality service to our clients” (Clinical Officer) | 14 | 21 |
| Ensure the objectives are achieved | “You know, in every organization, you must have what we call objectives or goals. So, as a leader, you must work so that you achieve those goals and objectives. And by working... When you want to achieve those goals, you have to make sure that everybody is involved. Like a leader, if you have a task, you identify an individual who can do that task and it can work” (Clinical Officer) | 5 | 6 |
| Give direction and take lead of a program | “So, according to me leadership is like giving the way forward for the people who are working and you are leading them to where you are supposed to go… healthcare system leadership is giving a way to the people who are working under you so that you achieve your objectives of treating the patients because in a healthcare system there are daily activities so you want like to achieve the objectives of the patients or solving the patients’ problems. So you give the way to the people who are under you so that they know their objectives and how to achieve their objectives” (Clinical Officer) | 13 | 17 |
| Link between the management and staff | “We have our in charge and most of the times she is the one who connects us with the management level, if we have issues, she is the one to take the issues upwards and if there is anything that has to be communicated again from the chief of party or from the clinical manager, she is the one to relay the information” (Clinical Officer) | 11 | 16 |
| Organize and facilitate trainings for the team members | “It could be we need to have trainings and he is the one who arranges. Number three is representing us in activities outside here maybe acquiring some skills outside there and then come back and train us. I think those are the most important that I see in our set-up… you understand whereby care providers, all of them, cannot go for training. For example, it is on gender-based violence, he can represent us then at the end of the day, come and brief us on what has been trained” (Clinical Officer) | 5 | 9 |
| Plan activities and services and budgeting | “Another key thing that my leader is doing is the planning and budgeting for the allocation of resources, the allocation of staff such that who is allocated to work where and at what time. When there are emergencies, he assigns who is going to intervene, what are the challenges and who is going to handle them” (Nurse, Female) | 5 | 5 |
| Provide mentorship to other staff | “And then secondly, there are policies, government policies like when they are rolling them down, she is the lead person, like she mentors us” (Clinical Officer) | 7 | 7 |
| Receives and provide reports | “Another role he does is also to collect data; to collect specific data and pertaining to the healthcare system, targets. He also reports this data, gets analyzed. You realize that with HIV there is so much data; those ones in care, those one who have defaulted, those to follow-up, and all that” (Clinical Officer) | 6 | 9 |
| Representation of a unit | “Another thing is representation of that unit because in as much as you are working as a team at that place, this leader at some point will have to be accountable for the area in which they are working on so that in case of any issues, you don't always have to fault others. So this particular leader is the one who will carry the burden of being accountable and being responsible for that particular area” (Clinical Officer) | 2 | 3 |
| Solve problems | “And some patients have complaints; the delays, I was not treated well, somebody is handled badly...A leader should come down to earth to settle down the issues. Sometimes you apologize...One of may have said something which is not good and hurts. A leader should come down to be able to apologize on behalf of that staff and clear the matter” (Nurse) | 10 | 10 |
| Supervisory role | “Number one is to do supervisory roles like daily supervision and to make sure people are at work and doing the right things and delivering. You know, implementing what they are supposed to be doing” (Clinical Officer) | 11 | 16 |
| Collaborate with other departments | “The healthcare system being intertwined; the departments being intertwined. And you realize that one department cannot function on its own, you really have to do some coordination and consultations with other departments” (Clinical Officer) | 16 | 23 |
