# Supplementary material for: Leading from the bottom: The clinical leaders roles in an HIV primary care facility in Eldoret, Kenya
Source: PLoS One. 2024 May 31;19(5):e0302066. doi: 10.1371/journal.pone.0302066 (PMC11142606; doi:10.1371/journal.pone.0302066)
Supplement: Attachment — Submitted filename: Response to Reviewers.docx [file pone.0302066.s004.docx]

**Rebuttal Letter Addressing Reviewer’s Comments**

**Reviewer #1**

**Abstract.**

**Reviewer:** The authors report that “the study presents views of healthcare providers on strategic leadership roles of clinical leaders in an HIV primary facility in Eldoret”. It may remain unclear to the readers whether this was the intended purpose of the study or one of the findings, or something coincidental.

***Response: The purpose has been restructured “to explore the views of healthcare providers of what they perceived as clinical leader roles” rather than presenting the views.***

**Reviewer:** In the abstract, under results section, authors report that “the results identified 4 themes that explained the perceived clinical leader’s strategic roles… The way this is phrased makes one wonder what the source of these 4 themes was. Could it be from a literature search for example? Was it from the respondents? The statement needs to be rephrased and edited.

***Author Response: The four themes emerged from the study findings (interviews) and the related ones were grouped into a single theme (strategic, managerial, supervisorial and research).***

**Reviewer:** And what are the 4 themes identified about? Are they defining management style? Qualities of leaders? The structure and language should be revised so that any reader can easily understand what is being presented here.

***Author Response: The 4 themes reflect the leadership and management style in terms of the roles of clinical leaders in the HIV primary care facility. A revision has been made in the conclusion and some new literature has been added to enrich and address the flow of the concept in the background.***

**Reviewer:** The conclusion seems to be mixed up with recommendations. This should be corrected.

***Author Response: A concise conclusion statement has been provided that reflects the findings in the abstract and the main document, and is separated from the study recommendations.***

**Background**

**Reviewer:** In line 36 (the very first sentence under this section), is the recognition just in literature or in practice?

***Author Response: This statement has been revised to provide a holistic picture of the importance of clinical leadership in literature and practice, particularly in strengthening health systems and improving healthcare services***

**Reviewer:** In line 42, the authors state that “a study concluded that effective clinical leadership..... Is it the current study or some other study? By who? Revise the statement appropriately so as to flow well.

***Author Response: A clear write-up has been provided to ensure the flow of this statement. Also, additional literature has been added to back up the claim/argument and ensure a logical flow***

**Reviewer:** The purpose of the study and/or objectives seem not to appear anywhere in the main body.

***Author Response: Additional literature on the role of clinical leaders in different contexts has been added and the gap in the HIV context highlighted.***

**Methods**

**Reviewer:** It would be nice for the authors to give a justification for the study design used.

***Author Response: A revision has been done to justify the design; “to gain an in-depth understanding of the perceived clinical leadership roles” as perceived by care providers***

**Reviewer:** The authors ought to tell us more on how they arrived at the number of respondents (22); how they were selected, who they are and how representative the number was.

***Author Response: 25 of the healthcare providers working in the MTRH-AMPATH facility were approached individually to participate in the study. The study reached saturation at 22 upon consent. A purposive sampling was used and they were approached individually. The providers included clinical officers, nurses, counselors, and a pharmacist, who were present at the facility at the time of the study and consented to participate. Their views reflected the perceptions of other providers in the facility.***

**Reviewer:** The sub-section “Human subjects” in line 104 should be changed to Ethical considerations.

***Author Response: This has been changed and described after data analysis section.***

**Reviewer:** The authors ought to describe the data analysis process more precisely, in a way that sounds scientific and convincing. They should also tell who handled the analysis and what their expertise is in qualitative data analysis is.

***Author Response: This has also been revised e.g. from data transcription and management in Nvivo software, reading through the transcripts by all the authors, coding by 2 authors with qualitative expertise, codes and categories examined to identify patterns and themes. Also, reliability and validity were ensured and a final codebook was developed that informed the final write-up of the results”***

**Reviewer:** It is not clear whether the 5 themes were identified at the planning stage prior to the data collection or they emerged from the respondents during the data collection. And are they 5 or 4?

***Author Response: They are 4 themes that were identified from the respondents during data collection and were categorized into 4 major themes. This has been clearly indicated in the results section.***

**Findings**

**Reviewer:** If the themes are describing strategic roles of clinical leaders, I find it funny to see that the first theme itself is “strategic roles”. In other words, are all the themes describing strategic roles of leadership? If so, how can strategic roles itself be one of the themes then? You need to polish the language around this.

***Author Response: This has been revised from the topic to the conclusion of the study, to address clinical leadership roles in HIV primary care and the themes that describe their roles include (strategic, managerial, supervisory and research). This removes a conflict between the topic and the results.***

**Reviewer:** What are the abbreviations CO1, CO2, CO3, P1, NR1 supposed to mean? The authors should acquaint themselves with and/or adopt the common practice when it comes to giving quotes.

***Author Response: These abbreviations have been revised to conform with the common practice of giving quotes. CO1, means Clinical Officer, Participant 1. This has now been revised to read “Participant #1: Clinical Officer, Participant #2: Nurse, etc. to provide a uniform flow.***

**Reviewer:** Also note that while giving these voices/quotes to substantiate arguments in the findings section is expected, these quotes should not dominate the section and should only be given where necessary. It may not be necessary to give so many voices/quotes, like 3, 4 or more to support one point.

***Author Response: Some of the quotes that present the same information have been removed from the main document. They are 1 or 2 supporting the arguments (changes tracked).***

**Reviewer:** Is the findings section presenting findings or themes? You need to be clear on this. For example on page 10, line 220, the authors state that “a strong theme that emerged among the patients was......”. Are issues that emerged themes or findings? This needs to be corrected throughout the section.

***Author Response: The section has been revised to reflect “findings” and not “themes”, and applied to all the findings.***

**Discussion**

**Reviewer:** The discussion section needs to be revised so that it rhymes well with the findings section. In some of the parts, the coherence and smooth flow is lacking. Also there should be coherence and logical flow even as citations are being made within this section, not just to drop in apparently from nowhere. For example assertions in sentence 361-362, 398 – 399; 399 - 401 and several others seem to be hanging.

***Author Response: The discussion section has been revised to ensure coherence (changes tracked)***

**Reviewer:** The discussion section has lots of very long sentences, that need to be revised and broken/shortened appropriately. For example single sentences like the one from line 347 to line 350, 371 – 374, 395 – 398 and several others which are very long and without any break should be revised where possible. The reader can easily get lost along the way.

***Author Response: Long sentences have been revised to short sentences to ensure flow and readability (changes tracked).***

**Reviewer:** In line 398 – 399, the authors state that “Our findings agree with a study that a good mentor empowered, encouraged, and inspired the novice (42)”. What is this supposed to mean? This and other similar, apparently confusing statements should be revised.

***Author Response: This section has been revised to provide a clear explanation e.g. “the study concurs with previous literature that physician leaders value the role of mentorship because they believed that they are natural mentors, and they believed that they have reached their current position because of someone who mentored them” (changes tracked).***

**Strength and limitations**

**Reviewer:** The qualitative approach to data collection itself is just a methodology and doesn’t guarantee strength of a study. It is not obvious that a qualitative approach to data collection automatically ensure rigor. The authors should therefore tell us more about strength of their study, as well as the weakness.

***Author Response: The strengths and limitations have been revised, e.g. “To our knowledge, there has been little information published on the role of clinical leaders in HIV primary care facilities. Hence, the findings of this study provide rich-based views and experiences of providers that shed more light on the varied roles of clinical leaders at the primary care facility level. The findings also provide important aspects for facility management decision-making on quality improvement of service delivery in the HIV primary care setting. While we believe this is a novel study in the HIV context, some limitations are important to acknowledge. First, clinical leaders and patients were not interviewed who could have provided different perspectives on clinical leader roles and this demands a study to obtain their views to gain an understanding of issues surrounding being a clinical leader in an HIV care system. Secondly, the providers who were interviewed included clinical officers, nurses, counselors and a pharmacist, hence little representation of the range of experiences of other providers who were not captured in the study. Third, providers expressed their views and experiences concerning the perceived clinical leader roles in the present HIV facility, hence may have varied degrees of transferability in other settings with varied aspects such as geographical location and service delivery. Fourth, this study only focused on clinical leaders at the lower level of the organization and may be interesting to obtain views from other leaders in the hierarchy of the organization (meso and strategic levels). Fifth, there is a danger of social desirability bias since most of the statements made by the healthcare providers tended to be positive and this may not reflect a true representation of their views and behaviour because they were evaluating their clinical leader in charge. Also, the set of guiding questions did not focus on relationship dynamics that relate to trust and hierarchies within the care system”.***

**Reviewer:** They could also find a better conclusion, given the study findings.

***Author Response: The conclusion has also been revised e.g. “This study explored the roles of clinical leaders in an HIV primary care facility. The study established a range of leadership roles that clinical leaders perform including strategic leadership roles, managerial leadership roles, supervisorial leadership roles and research roles. The findings, therefore, highlight that clinical leaders at the microsystem level perform extraordinary leadership roles that impact HIV care, service delivery and health system performance. The findings also inform the management of health facilities of the important roles of clinical leaders that are relatively similar or different from those performed by leaders at different levels in the health facility, hence informing a future research gap. Hence, they should build the capacity of clinical leaders and review standard operating procedures to include key strategic decisions at all levels to maximize clinicians' contribution to HIV care and responsive health systems.***

**Reviewer:** The study addresses an area that is of relevance and critical for improvement of health service delivery. However, the manuscript needs to be re-organized, and be some language editing be done so as to flow well.

***Author Response: The manuscript has been re-organized and revised. Thank you.***

**Reviewer #2**: “Strategic” is overused and used in different in contexts. As this is a key term, please be more precise in what is encapsulated in “strategic.” This definition comes out slightly in the results but feels a but tautological to state that theme for perceived clinical leader’s strategic roles was being strategic (lines 19-20)

***Author response: The word strategic has been corrected to address the finding under the strategic leadership roles only. It has been removed in the other findings (middle-management and supervisory), to ensure uniformity and avoid redundancy use.***

**Reviewer:** I am surprised the issue of hierarchy and trust (as well as other social dynamics) did not come more prominently in the findings and themes, and perhaps this reflects also potential social desirability bias or some considerations on reflexivity on what health workers are willing to disclose with regard to their “clinical leaders” Moreover, the findings/themes seem very basic and what you would expect to find on a terms of reference. There should be a re-analysis of themes to identify more profound findings that can add to the literature.

***Author response: First the interview guide did not pose questions on aspects of relational dynamics (trust and hierarchy) and after re-analyzing the themes to check if any of the aspects emerged, there were not established. Therefore, this has been acknowledged as a social desirability bias in the limitation section.***

**Reviewer:** It is also not as clear as how the themes of strategic, managerial and supervisory are distinct themes. The themes should be better fleshed out to describe what strategic means and its facets.

***Author response: The themes have been reorganized into strategic, middle-level and supervisory. These describe the hierarchy of leadership in an organization and it shows that clinical leaders perform roles that cut across the leadership hierarchies in a care system.***

**Reviewer:** It would also be interesting to overlay the background with literature on “supportive supervision” and “in-service training/mentorship.” This comes out in the discussion, but it’s not clear in the background how this is different and what value strategic leadership means over existing forms support.

***Author response: Literature on supportive supervision and mentorship has been added in the background of the study.***

**Reviewer:** In line 76-77, there is not really context for who respondents were acknowledging that it is a grey area.

***Author response: This statement has been revised to capture the aspect of the gap. “grey area for development because their practice was still developing and involves responsibility for the practice of others”***

**Reviewer:** The strengths and limitations should also include the potential biases, including social desirability bias, especially since most of the quotes and what was stated tended to be positive. Acknowledging that health workers are providing this feedback about their supervisors in the context of their work could put some limitations on the extent of information that would be shared.

***Author Response: A statement on the danger of social desirability bias has been included to take care of the positive statements made by the care providers.***
