# Supplementary material for: Leading from the bottom: The clinical leaders roles in an HIV primary care facility in Eldoret, Kenya
Source: PLoS One. 2024 May 31;19(5):e0302066. doi: 10.1371/journal.pone.0302066 (PMC11142606; doi:10.1371/journal.pone.0302066)
Supplement: Attachment — Submitted filename: Response to Reviewers_second review_Jan 2024.docx [file pone.0302066.s006.docx]

**Rebuttal Letter Addressing Reviewer’s Comments**

**Reviewer #1**

**Second Review January 2024**

Reviewer’s comments (R1)

1. Comment 2, made during the first review has not been addressed. “The results identified4 themes” is not grammatically right. The phrase therefore needs to be revised.

**Response: We have addressed this comment in the abstract and results sections.**

***In the abstract: “The following themes were identified from the analysis regarding perceived clinical leaders’ roles in an HIV primary care facility: 1) Research roles….***

***In the results section: “Participants described four themes that characterize clinical leadership roles in an HIV primary care facility: strategic leadership roles, middle-management leadership roles, supervisory leadership roles and research roles”***

2. Another comment asked for the purpose of the study/objectives to be included. This hasn’t been addressed. Instead, the authors have added literature, making the background section longer.

***Response:***

***At the end of the background, we have included “*Our study explored the perspectives of healthcare providers on the leadership roles of clinical leaders in an HIV primary care facility in Eldoret, Kenya.**

***We have also included the purpose in the background of the study and summarized text to reflect the gap and the scope of the study.***

3. The authors should give a justification for the study design used.

***Response: A qualitative exploratory study was conducted between December 2019 to May 2020 involving in-depth interviews to explore the views of healthcare providers to obtain an in-depth understanding of the perceived leadership roles of clinical leaders that may influence patient outcomes and health system improvements.***

4. It would be nice to have a detailed description of the study participants and how they were sampled, in the relevant section. This is for the readers, not the reviewer.

***Response: We have separated the sections in the methods to highlight: study design, study setting, participant sampling and recruitment, data collection procedures, data management and analysis to provide clarity and coherence in the description***

***In the ‘participant sampling and recruitment section’ we described as follows;***

***“The participants in our study involved frontline healthcare providers (clinicians, nurses, counselors, and a pharmacist). The total population of healthcare providers at MTRH-AMPATH was 50 at the time of the study. We first presented the research permits for conducting the study in AMPATH to the clinical leaders in charge of various clinics to explain the purpose of the study and obtain approval to interview the HCPs. We purposively and conveniently approached 25 HCPs individually and 22 consented to participate in in-depth interviews. Literature suggests that a sample size of 10 is adequate if a homogenous group of people is interviewed, nevertheless, a sample size of 15-30 is considered appropriate for a qualitative study (22)”.***

5. The data analysis section is unconvincing. First, the authors state that data was managed in Nvivo software. Which version of Nvivo? And what exactly was done using Nvivo? Then what is subsequently stated doesn’t seem to suggest that there was any computer-assisted data analysis. It appears as if the coding and analysis was done manually, but the authors feel obliged to mention Nvivo. The authors need to tell what exactly was done for analysis. Also note that you can’t increase the quality of the data by just counterchecking codes. The quality of the data is determined at the point of data collection.

***Response: “Thematic analysis was used to analyze qualitative data (23,25). The first author began by transcribing the audio recordings verbatim then the transcripts were read and re-read by all the authors to familiarize themselves with the content of the data and understand its richness and diversity. The transcribed data was imported to NVivo12 for management and coding. Two authors with qualitative backgrounds coded the data, compared, discussed, and examined the codes and categories to identify patterns and themes for interpretation. All the authors then read and discussed the themes and agreed on the final themes to be included in the report. A final codebook indicating emerging themes was then developed that informed the final write-up of the results and supported by relevant quotes from the data. To ensure validity and reliability, and increase the quality of the data, the codes were counterchecked and appraised by all authors to confirm there was no repeated and conflicting content (26,27). The data collection procedures and analysis were made clear to help maintain consistency and rigor throughout the study. Team debrief sessions were held during the data collection to enhance the quality of the data. The analysis informed descriptive categories/domains that represent thematic healthcare providers' perceptions of the clinical leadership roles of clinical leaders in an HIV primary care facility”.***

6. The manuscript still requires some language editing.

***Response: We have reviewed the entire manuscript and made necessary edits to ensure coherence in language and general flow***
