# Supplementary material for: Leading from the bottom: The clinical leaders roles in an HIV primary care facility in Eldoret, Kenya
Source: PLoS One. 2024 May 31;19(5):e0302066. doi: 10.1371/journal.pone.0302066 (PMC11142606; doi:10.1371/journal.pone.0302066)
Supplement: Attachment — Submitted filename: Rebuttal Letter Addressing Reviewer and editors comments.docx [file pone.0302066.s008.docx]

**Rebuttal Letter Addressing Reviewer’s and Editor’s Comments**

Journal Requirements:

Please review your reference list to ensure that it is complete and correct. If you have cited papers that have been retracted, please include the rationale for doing so in the manuscript text, or remove these references and replace them with relevant current references. Any changes to the reference list should be mentioned in the rebuttal letter that accompanies your revised manuscript. If you need to cite a retracted article, indicate the article’s retracted status in the References list and also include a citation and full reference for the retraction notice.

**Response:** The reference list has been reviewed to ensure it is updated. 1 journal article article **of “**Oates K. The new clinical leader. Vol. 48, Journal of Paediatrics and Child Health. 2012. p. 472–5.: / which was initially cited in APA format has now been cited in the current citation format and included as citation No.37 in the body document and reflected in the reference list. Retracted papers are not applicable in the references used in this document.

[Note: HTML markup is below. Please do not edit.]

Reviewers' comments:

Reviewer's Responses to Questions

**Comments to the Author**

1. If the authors have adequately addressed your comments raised in a previous round of review and you feel that this manuscript is now acceptable for publication, you may indicate that here to bypass the “Comments to the Author” section, enter your conflict of interest statement in the “Confidential to Editor” section, and submit your "Accept" recommendation.

Reviewer #1: All comments have been addressed

Reviewer #2: All comments have been addressed

2. Is the manuscript technically sound, and do the data support the conclusions?

The manuscript must describe a technically sound piece of scientific research with data that supports the conclusions. Experiments must have been conducted rigorously, with appropriate controls, replication, and sample sizes. The conclusions must be drawn appropriately based on the data presented.

Reviewer #1: Yes

Reviewer #2: Partly

3. Has the statistical analysis been performed appropriately and rigorously?

Reviewer #1: N/A

Reviewer #2: Yes

4. Have the authors made all data underlying the findings in their manuscript fully available?

The [PLOS Data policy](http://www.plosone.org/static/policies.action#sharing) requires authors to make all data underlying the findings described in their manuscript fully available without restriction, with rare exception (please refer to the Data Availability Statement in the manuscript PDF file). The data should be provided as part of the manuscript or its supporting information, or deposited to a public repository. For example, in addition to summary statistics, the data points behind means, medians and variance measures should be available. If there are restrictions on publicly sharing data—e.g. participant privacy or use of data from a third party—those must be specified.

Reviewer #1: No

Reviewer #2: Yes

5. Is the manuscript presented in an intelligible fashion and written in standard English?

PLOS ONE does not copyedit accepted manuscripts, so the language in submitted articles must be clear, correct, and unambiguous. Any typographical or grammatical errors should be corrected at revision, so please note any specific errors here.

Reviewer #1: Yes

Reviewer #2: Yes

6. Review Comments to the Author

Please use the space provided to explain your answers to the questions above. You may also include additional comments for the author, including concerns about dual publication, research ethics, or publication ethics. (Please upload your review as an attachment if it exceeds 20,000 characters)

Reviewer #1: No further comments. The authors have fully addressed all concerns earlier raised; and i am happy with the submission.

Reviewer #2: The distinctions in the categories between Middle- Level Leadership and Supervisory roles are not always clear. Perhaps it would be better to organize the themes around functions, such as "establishing linkages between health system levels," supervision, etc.

- I have reorganized this section to provide a sub-title “ Interconnecting health systems and supervisory oversight roles” that would describe the themes around the different leadership levels of the health systems. I have provided a brief introduction of the section to reflect the interconnectedness and the supervisory oversight roles of the clinical leader in the health system.
- This is the brief introduction “Participants reported that clinical leaders play an important role in interconnecting different levels of health systems management by facilitating communications, collaboration, and coordination among various stakeholders. Through effective supervisory oversight, the leaders ensure the delivery of high-quality HIV care while fostering professional growth and development within their teams.
- The emerging themes and quotes around interconnectedness and supervisory oversight have been reorganized to fit the 2 sub-themes.
- The reorganization of the themes has been reflected in the results section, the discussion section to enhance a good flow of the themes, and the sub-title revised “interconnecting health systems and supervisory oversight’ in the abstract to reflect 3 roles of a clinical leader.

7. PLOS authors have the option to publish the peer review history of their article ([what does this mean?](https://journals.plos.org/plosone/s/editorial-and-peer-review-process#loc-peer-review-history)). If published, this will include your full peer review and any attached files.


If you choose “no”, your identity will remain anonymous but your review may still be made public.


**Do you want your identity to be public for this peer review?** For information about this choice, including consent withdrawal, please see our [Privacy Policy](https://www.plos.org/privacy-policy).

Reviewer #1: No

Reviewer #2: No
