# Supplementary material for: Leading from the bottom: The clinical leaders roles in an HIV primary care facility in Eldoret, Kenya
Source: PLoS One. 2024 May 31;19(5):e0302066. doi: 10.1371/journal.pone.0302066 (PMC11142606; doi:10.1371/journal.pone.0302066)
Supplement: Attachment — Submitted filename: Reviewer comments.docx [file pone.0302066.s003.docx]

**Reviewer’s comments**

**Abstract.**

The authors report that “the study presents views of healthcare providers on strategic leadership roles of clinical leaders in an HIV primary facility in Eldoret”. It may remain unclear to the readers whether this was the intended purpose of the study or one of the findings, or something coincidental.

In the abstract, under results section, authors report that “the results identified 4 themes that explained the perceived clinical leader’s strategic roles... The way this is phrased makes one wonder what the source of these 4 themes was. Could it be from a literature search for example? Was it from the respondents? The statement needs to be rephrased and edited.

And what are the 4 themes identified about? Are they defining management style? Qualities of leaders? The structure and language should be revised so that any reader can easily understand what is being presented here.

The conclusion seems to be mixed up with recommendations. This should be corrected.

**Background**

In line 36 (the very first sentence under this section), is the recognition just in literature or in practice?

In line 42, the authors state that “a study concluded that effective clinical leadership..... Is it the current study or some other study? By who? Revise the statement appropriately so as to flow well.

The purpose of the study and/or objectives seem not to appear anywhere in the main body.

**Methods**

It would be nice for the authors to give a justification for the study design used.

The authors ought to tell us more on how they arrived at the number of respondents (22); how they were selected, who they are and how representative the number was.

The sub-section “Human subjects” in line 104 should be changed to Ethical considerations.

The authors ought to describe the data analysis process more precisely, in a way that sounds scientific and convincing. They should also tell who handled the analysis and what their expertise is in qualitative data analysis is.

It is not clear whether the 5 themes were identified at the planning stage prior to the data collection or they emerged from the respondents during the data collection. And are they 5 or 4?

**Findings**

If the themes are describing strategic roles of clinical leaders, I find it funny to see that the first theme itself is “strategic roles”. In other words, are all the themes describing strategic roles of leadership? If so, how can strategic roles itself be one of the themes then? You need to polish the language around this.

What are the abbreviations CO1, CO2, CO3, P1, NR1 supposed to mean? The authors should acquaint themselves with and/or adopt the common practice when it comes to giving quotes.

Also note that while giving these voices/quotes to substantiate arguments in the findings section is expected, these quotes should not dominate the section and should only be given where necessary. It may not be necessary to give so many voices/quotes, like 3, 4 or more to support one point.

Is the findings section presenting findings or themes? You need to be clear on this. For example on page 10, line 220, the authors state that “a strong theme that emerged among the patients was......”. Are issues that emerged themes or findings? This needs to be corrected throughout the section.

**Discussion**

The discussion section needs to be revised so that it rhymes well with the findings section. In some of the parts, the coherence and smooth flow is lacking. Also there should be coherence and logical flow even as citations are being made within this section, not just to drop in apparently from nowhere. For example assertions in sentence 361-362, 398 – 399; 399 - 401 and several others seem to be hanging.

The discussion section has lots of very long sentences, that need to be revised and broken/shortened appropriately. For example single sentences like the one from line 347 to line 350, 371 – 374, 395 – 398 and several others which are very long and without any break should be revised where possible. The reader can easily get lost along the way.

In line 398 – 399, the authors state that “Our findings agree with a study that a good mentor empowered, encouraged, and inspired the novice (42)”. What is this supposed to mean? This and other similar, apparently confusing statements should be revised.

**Strength and limitations**

The qualitative approach to data collection itself is just a methodology and doesn’t guarantee strength of a study. It is not obvious that a qualitative approach to data collection automatically ensure rigor. The authors should therefore tell us more about strength of their study, as well as the weakness.

They could also find a better conclusion, given the study findings.

The study addresses an area that is of relevance and critical for improvement of health service delivery. However, the manuscript needs to be re-organized, and be some language editing be done so as to flow well.
