# Supplementary material for: Leading from the bottom: The clinical leaders roles in an HIV primary care facility in Eldoret, Kenya
Source: PLoS One. 2024 May 31;19(5):e0302066. doi: 10.1371/journal.pone.0302066 (PMC11142606; doi:10.1371/journal.pone.0302066)
Supplement: Attachment — Submitted filename: Reviewer comments PLOS (R1).docx [file pone.0302066.s005.docx]

Reviewer’s comments (R1)

1. Comment 2, made during the first review has not been addressed. “The results identified4 themes” is not grammatically right. The phrase therefore needs to be revised.
2. Another comment asked for the purpose of the study/objectives to be included. This hasn’t been addressed. Instead, the authors have added literature, making the background section longer.
3. The authors should give a justification for the study design used.
4. It would be nice to have a detailed description of the study participants and how they were sampled, in the relevant section. This is for the readers, not the reviewer.
5. The data analysis section is unconvincing. First, the authors state that data was managed in Nvivo software. Which version of Nvivo? And what exactly was done using Nvivo? Then what is subsequently stated doesn’t seem to suggest that there was any computer-assisted data analysis. It appears as if the coding and analysis was done manually, but the authors feel obliged to mention Nvivo. The authors need to tell what exactly was done for analysis. Also note that you can’t increase the quality of the data by just counterchecking codes. The quality of the data is determined at the point of data collection.
6. The manuscript still requires some language editing.
