# Supplementary material for: Leading from the bottom: The clinical leaders roles in an HIV primary care facility in Eldoret, Kenya
Source: PLoS One. 2024 May 31;19(5):e0302066. doi: 10.1371/journal.pone.0302066 (PMC11142606; doi:10.1371/journal.pone.0302066)
Supplement: Attachment — Submitted filename: Reviewer comments.docx [file pone.0302066.s007.docx]

Reviewer comments

All concerns raised earlier have been addressed and am happy with the submission.
